# Case Report: Cytodiagnosis of pigmented villonodular synovitis involving carpal bones of right wrist

**DOI:** 10.12688/f1000research.141797.1

**Published:** 2023-11-21

**Authors:** Dr. Shreya Giri Goswami, Dr. Arvind Bhake, Dr. Simran Khan, Dr. Soumya Agrawal, Dr. Jayashree Bhawani

**Affiliations:** 1 Department of Pathology, Datta Meghe Institute of Higher Education and Research, Sawangi, Wardha, Maharashtra, 442001, India

**Keywords:** Pigmented Villonodular Synovitis, Fine Needle Aspiration Cytology, Cytomorphology, Multiosteotic involvement, Carpal bones

## Abstract

**Background:**

Pigmented Villonodular Synovitis (PVNS) is a rare disease of osteoskeletal tissue. Cytodiagnosis of PVNS on fine needle aspiration (FNA) smears is therefore rarely reported. The PVNS usually affects the larger joints. The involvement of the smaller joints and bones are uncommon.

**Case presentation:**

The reported case is one such rarity wherein the diagnosis of PVNS was carried out on the FNAC. The case showed the involvement of all carpal bones except for the pisiform. The 2 ^nd^- 5 ^th^ metacarpal bases were also found to be involved in the disease process. The presence of sheets of synoviocytes with brown altered hue to the cytoplasm along with multinucleate giant cells and pigmented macrophages were characteristically present in the smears of FNA. The diagnosis was confirmed on the tissue biopsy. The present case is reported for its unusual multiosteotic involvement of wrist joint bones and the metacarpal bones simultaneously with radiological evidence. The cytomorphology of the lesion in the present case were noteworthy as a learning experience in reporting of PVNS of wrist joint on FNA smears.

## Introduction

Pigmented Villonodular Synovitis (PVNS) is an uncommon lesion in the practice of orthopedics. Lesion of PVNS is dominantly localized around the large joints, especially of the knee. Its occurrence at the small joints such as the wrist, and interphalangeal region have also been reported in the literature but rarely. PVNS becomes a diagnostic dilemma because of its clinical presentation and deceivious radiological appearance. This is due to the infiltrative character of the lesion and its propensity to destroy the structure it involves. ^1^^–^^3^

Rare case reports of PVNS exist in the literature. A single large retrospective multicentric study of 237 cases of Xie *et al* ^1^ elaborated on the radiograph, MRI, and histopathological appearances of PVNS however, this study lacks a description of the cytodiagnosis of PVNS. This study further concludes that PVNS on many occasions remain clinically and radiologically unsuspected.

The fine needle aspiration cytology (FNAC) of the lesions affecting the joints, periarticular tissue, and bone has by now become the part of diagnostic algorithm. The FNAC in the diagnosis of PVNS has rarely been described in the literature, more so, the multiosteotic involvement by PVNS and its diagnosis by FNAC has also not been published in the literature. The present article reports one such rare case of PVNS of the wrist joint affecting all its bones along with the involvement of metacarpal bones. The report also describes the FNAC diagnosis of PVNS involving the bones of wrist joint along with the clinical presentation and radio imaging findings of the unusual case.

## Case report

**Figure 1.**
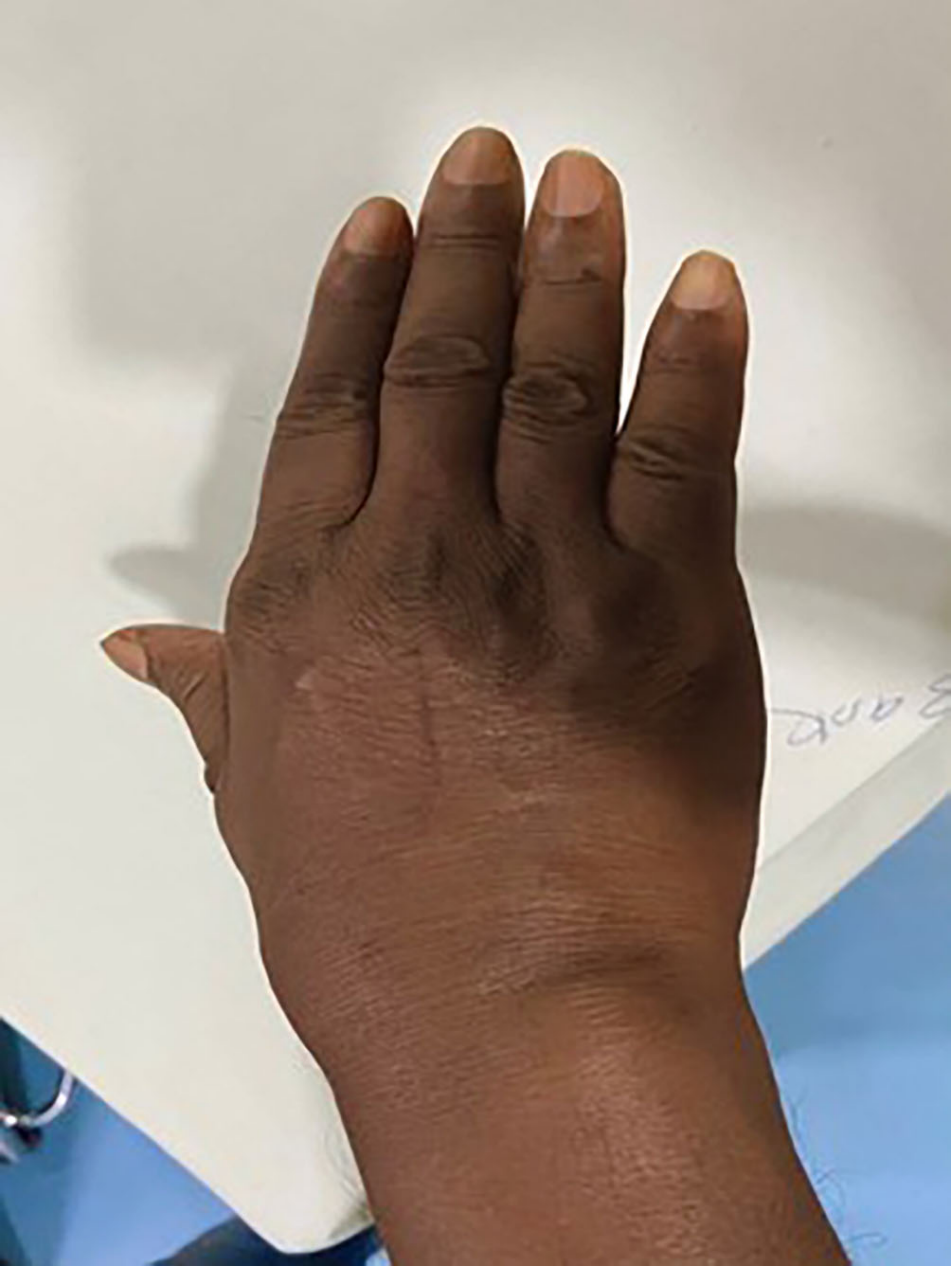
Photograph showing swelling on the extensor aspect near right wrist.

A 37-year-old male visited the outpatient department of orthopedics with complaints of pain and swelling in the right wrist that has lasted for two years ( Figure 1). At the outset, the pain was sudden and had a dull, aching quality. However, it gradually became continuous and characterized by a throbbing sensation. The throbbing pain in the wrist joint was existing for six months. The pain was associated with swelling. The swelling on the wrist joint too was progressive. The patient complained of aggravation of pain on movement and relieved on immobilization and rest. The pain reported by the patient in the wrist joint is non-radiating type. Patient has reported no history of locoregional trauma or fall. The history revealed no constitutional symptoms, fever, evening rise of temperature and loss of appetite. Patient provided a previous medical history of seeking consultation from a private healthcare provider who prescribed oral NSAIDs for pain relief. Patient gave no significant family, psycho-social or genetic history. He has not undergone any intervention for his complaints.

The locoregional examination of right wrist showed local swelling on the extensor as well as the supinator surface but more on former. The swelling was extending on the extensor surface of the palm. The swelling was non-pitting type and painful on minimal pressing. The clinical examination showed restricted joint movement and pain during movement. The local temperature of the swelling was not raised and it was negative for the transillumination test. There was no crackling sound on palpation in the swelling on the wrist. The other joints in the body were normal for their movements.

The rest of the physical and general examination were within normal limits. The systemic examination was normal. No organomegaly could be detected. The skin over the wrist on local examination was free of scar and sinuses but the skin overlying the swelling was tense. The radial artery on the wrist joint was palpable and normal for beats.

The complete blood count was carried out. It did not reveal any significant abnormality. The coagulation profile of APTT and prothrombin time too were normal. The electrolyte levels were also found within normal limits. The urea level was 27 and creatinine was 0.6% suggesting the normal kidney function test. The values of liver function test i.e. ALT, AST and ALP were within normal range. The total protein was 7.3 and total bilirubin was 0.7. The HIV testing was non-reactive.

**Figure 2.**
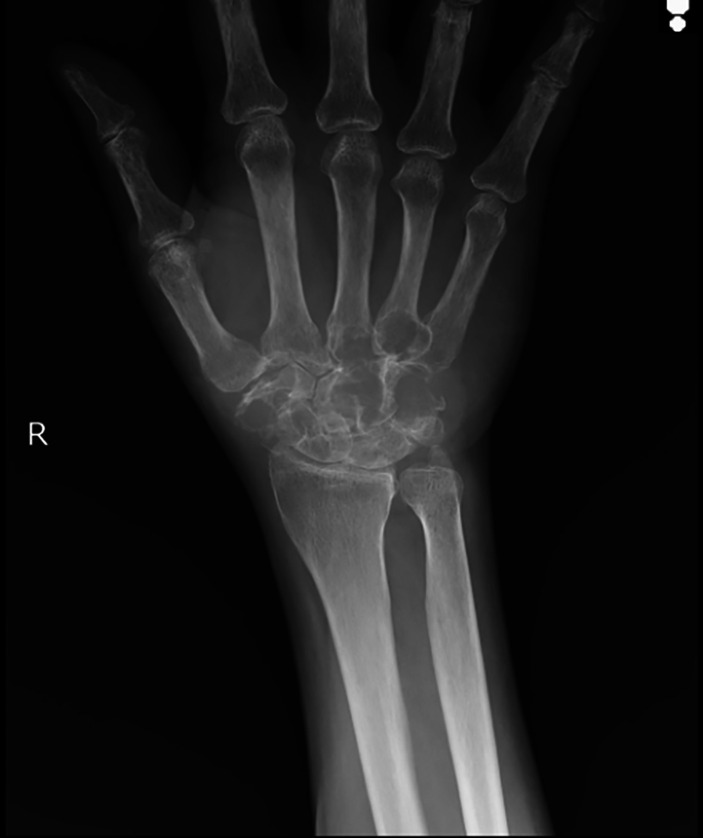
Anteroposterior X-ray right wrist demonstrated lytic lesions of carpal bones and base of 3 ^rd^ and 4 ^th^ metacarpal bones.

The patient was sent for X-ray right wrist and for an anteroposterior and lateral view. The X-ray showed lytic lesions of carpal bones of right wrist and lytic lesions of the base of 3 ^rd^ and 4 ^th^ metacarpals ( Figure 2). The diagnosis of involvement of bone by Primary Lymphoma was offered on the X-ray finding. The patient underwent MRI right wrist which showed ill-defined moderately hypointense signals involving all the carpal bones except for pisiform bone. MRI also showed involvement of the base of 2 ^nd^ to 5 ^th^ metacarpals bones with minimal expansion and thinning of adjacent cortices and subtotal obliteration of intercarpal joint. The diagnosis offered on MRI findings was Giant Cell tumour and Primary Lymphoma of carpal bones.

**Figure 3.**
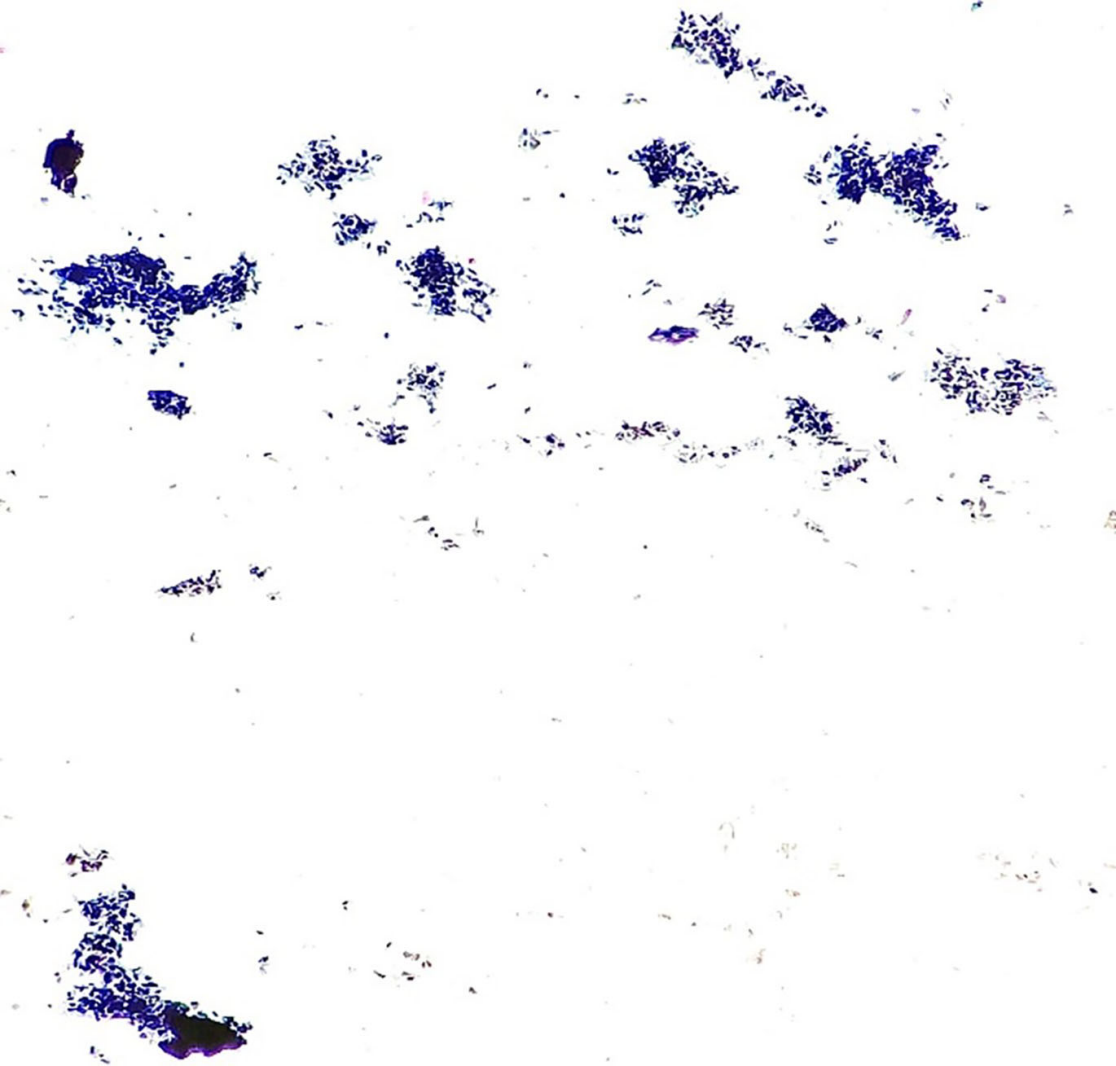
Photomicrograph FNAC – PVNS: Smear showed small isolated groups and sheets of synoviocytes (Papanicolaou Stain, 4×).

**Figure 4.**
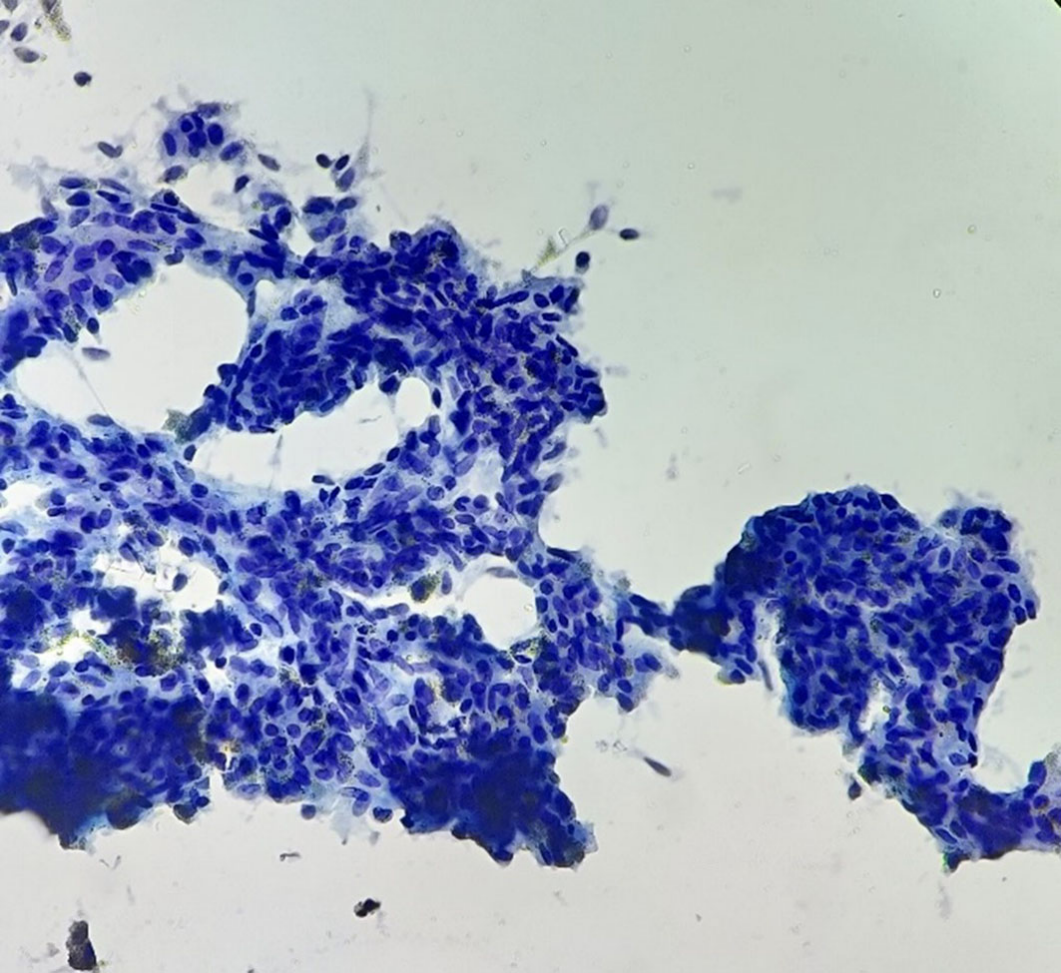
Photomicrograph FNAC – PVNS: Smear showed a cellular fragment of elongated spindled nuclear cells. The cytoplasmic pigments noticed within the sheets of synoviocytes. Few pigment-laden macrophages and stromal fragments were also seen (Papanicolaou Stain, 40×).

**Figure 5.**
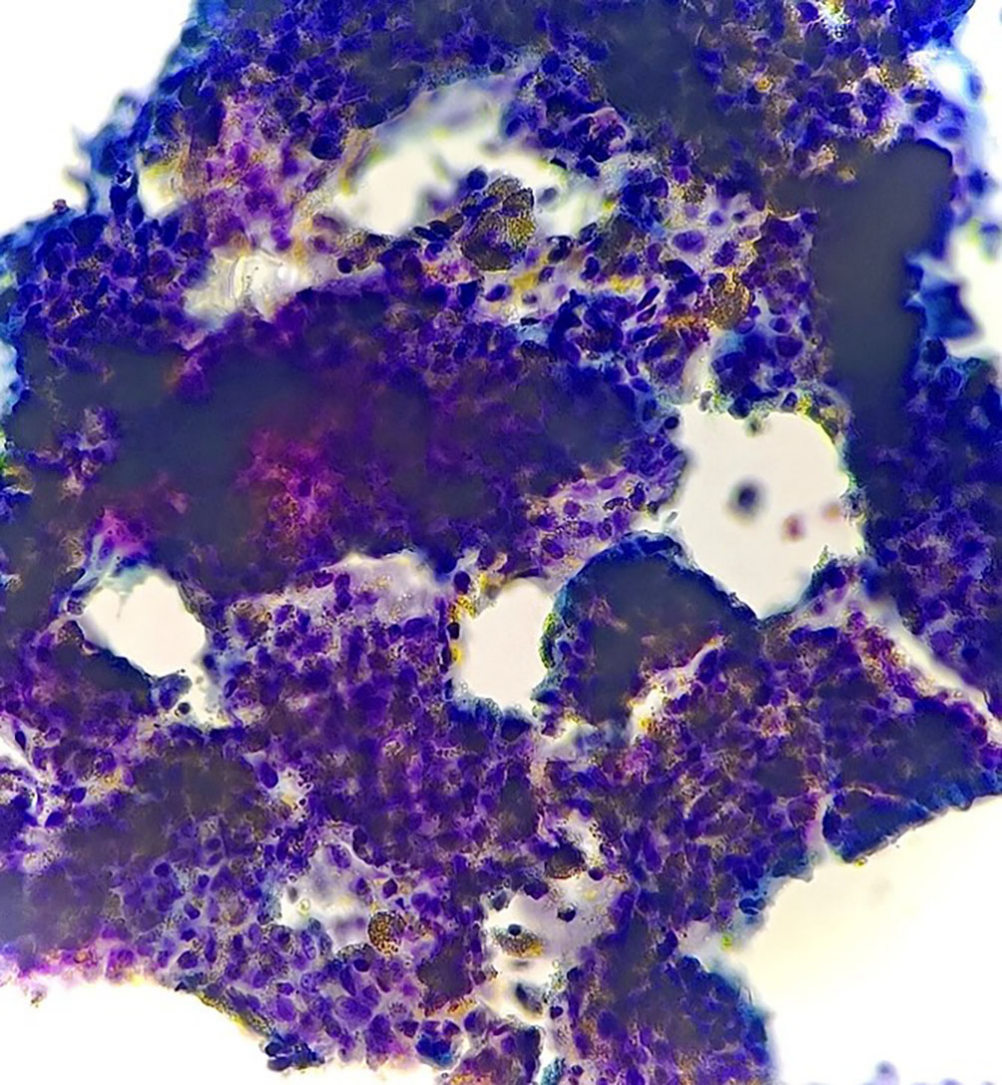
Photomicrograph FNAC – PVNS: Cytology smear showed macrophages containing pigments which were dark and brown. Rare place showed multinucleate giant cells (Papanicolaou Stain, 40×).

The call for FNAC from right wrist was received. It was carried out under the radio imaging guidance from two carpal bones involved in the process of disease and one metacarpal bone. The smears of FNAC showed isolated groups, sheets and cellular fragments of synoviocytes with little enlarged nuclei and frequent binucleation. There were small cellular fragments of elongated spindled nuclear cells. Also seen were isolated pigment-laden macrophages which were dark and brown. Multinucleate giant cells, lymphocytes, and rare stromal fragments too were seen ( Figures 3- 5). The FNAC diagnosis of Pigmented Villonodular Synovitis was offered.

The patient underwent a tru-cut biopsy from one of the involved carpal bones. The histopathological diagnosis was consistent with the diagnosis of PVNS.

## Discussion

Pigmented Villonodular synovitis is a slow growing benign and locally invasive tumor which is relatively uncommon. It is part of a family of lesions believed to arise from synovium of joints, tendons and bursae. ^2^^,^^3^ These lesions commonly occur around the large joints such as knee but the involvement of the bones and joints at atypical and rare sites have also been reported in the literature. ^4^^–^^6^

One such case report of involvement of the temporomandibular joint by PVNS has been reported by Lu *et al.* ^6^ PVNS is known to be affecting both genders and its average age of diagnosis is between 30–40 years. ^2^^,^^6^^,^^7^ One of the large retrospective multicentre studies of 237 cases of PVNS published by Xie *et al* ^1^ reports knee and hip as the commonest sites of occurrence of PVNS.

The present case of PVNS was of 37-year-old male which is the common age range for clinical presentation of PVNS and is also reported by few other studies. ^7^^,^^8^

The diagnosis of PVNS even on MRI is not straightforward as has been reported in the studies of Nair *et al*, Irisarri *et al*, Bhatnagar *et al*, Luo *et al* and Aldakhil *et al.* ^3^^,^^5^^,^^7^^,^^9^^,^^10^ One of the common differential diagnoses that was offered in MRI in the aforesaid studies was of Giant Cell Tumor of the tendon sheath irrespective of the site of occurrence.

In the present case, one of the differential diagnoses on MRI was Giant Cell Tumor of tendon sheath and involvement by Non-Hodgkin’s Lymphoma. Multiosteotic involvement by PVNS is scarcely reported in the literature.

Most of the studies report single joint bone involvement. Study by Luo *et al* ^9^ reported atypical bilateral PVNS of the wrist in a 14 years old boy. Luo *et al* ^9^ in their review summarized 26 studies on 30 adolescent patients with location of PVNS was knee followed by hip and ankle.

The present case is unique for the rarity of presentation wherein all the bones of the right wrist except pisiform bone and 2 ^nd^, 3 ^rd^, 4 ^th^ metacarpal bones were involved in the disease process. Such a rare occurrence of involvement of PVNS locally in the wrist bone along with involvement of metacarpal bones has not been reported in the search literature except for Luo *et al* ^9^ and Carpentiro *et al.* ^11^ There are very few case reports which describe the diagnosis of PVNS on FNAC i.e., Lu *et al*, Bhatnagar *et al*, Luo *et al* and Choi *et al*. ^6^^,^^7^^,^^9^^,^^12^

The FNAC of PVNS usually is brown coloured turbid aspirate. The present case which underwent FNAC at three sites yielded brown colour turbid fluidy material. This characteristic appearance of the aspirate of PVNS has also been shared in the article of Sitati *et al*. ^13^

Lu *et al* ^6^ made the cytodiagnosis of PVNS. The features this study elaborated in the diagnosis of PVNS were as follows; i) Moderately cellular smears. ii) Predominant population of mononuclear polygonal to elongated cells arranged in loosely cohesive clusters and singly. iii) Eccentric nuclear location. iv) Granular chromatin with rare nuclear grooves and distinct nucleoli. v) These cells cytoplasm contained hemosiderin granules, numerous multinucleate giant cells, and scant binucleate cells. The multinucleate giant cells carry 4 to 5 nuclei within it and were morphologically similar to the mononuclear cells.

Bhatnagar *et al* ^7^ made the cytodiagnosis of PVNS as their smears of FNAC contained proliferating synoviocytes placed in tight cohesive clusters of benign morphology. These cells were round carrying regular nuclei with bland chromatin, inconspicuous nucleoli and ill-defined granular cytoplasm. Another diagnostic component of this study was multinucleate giant cells along with singly scattered plump cells having oval pale nuclei with bland chromatin. Smears also show foamy macrophages, of which few were hemosiderin pigmented.

Luo *et al* ^9^ observed a few short spindle cells and a few inflammatory cells in the aspirate smears of PVNS.

The observations for the cytodiagnosis of PVNS for present case are similar to the one made by Lu, Bhatnagar and Luo *et al*. ^6^^,^^7^^,^^9^

The subsequent biopsy from the lesion of the present case showed histomorphology consistent with the diagnosis of PVNS.

## Conclusion

The pre-operative diagnosis of PVNS on FNAC can be achieved by the features of marked proliferative sheets of synoviocytes which are polygonal in shape and show cytoplasmic brown altered hue, multinucleate giant cells with the pigments, isolated macrophages which are hemosiderin pigmented along with background paucicellular population of lymphocytes.

The radio imaging investigations of X-ray, computed tomography (CT), and magnetic resonance imaging (MRI) findings help in asserting the cytodiagnosis of PVNS.

The present case is unique in multiple contexts. PVNS involving multiple small bones of the wrist and metacarpal bone being diagnosed on FNAC is rarely reported in the literature. The acquaintance of cytomorphological features of PVNS is of great added value as it guides on to the treatment of the patients of PVNS. This case is shared for its clinical, radiological, and cytopathological features with the practicing fraternity of orthopedics and pathology for its unique encounter.

## Consent

Written informed consent has been obtained from both the patient and their family for the publication of the patient’s clinical details. They were fully informed about the purpose and scope of the publication, and their privacy will be protected.

## Data Availability

All data underlying the results are available as part of the article and no additional source data are required. Zenodo. CARE Checklist for Cytodiagnosis of pigmented villonodular synovitis involving carpal bones of right wrist. DOI: 10.5281/zenodo.8409739.

## References

[1] Ping XG Jiang N Xiang LC : Pigmented Villonodular Synovitis: A Retrospective Multicenter Study of 237 Cases. Assassi S, editor. *PLoS One.* 2015 Mar 23;10(3):e0121451. 10.1371/journal.pone.0121451 25799575 PMC4370558

[2] Zhao L Zhou K Hua Y : Multifocal pigmented villonodular synovitis in a child: A case report. *Medicine.* 2016 Aug;95(33):e4572. 10.1097/MD.0000000000004572 27537585 PMC5370811

[3] Nair D Samuel S Varkey S : Pigmented villonodular synovitis in a rare site. *J Orthop Assoc South Indian States.* 2022;19(1):33. 10.4103/joasis.joasis_3_22

[4] Kapoor C Jhaveri M Soni R : Pigmented Villonodular Synovitis of the Knee Joint: A Case Report. *Cureus.* 2016 Oct 4 [cited 2023 Aug 27];8:e816. 10.7759/cureus.816 Reference Source 27843734 PMC5101109

[5] Irisarri C Yañez CJ : Extended Pigmented Villonodular Synovitis of the Hand. *Rev Iberoam Cir Mano.* 2017 Nov;45(02):125–129. 10.1055/s-0037-1608646

[6] Lu DY Zhang L Apple SK : Fine needle aspiration of pigmented villonodular synovitis of the temporomandibular joint. *Diagn Cytopathol.* 2011 Jan;39(1):45–48. 10.1002/dc.21362 21162093

[7] Bhatnagar K : Pigmented Villonodular Synovitis of Thumb-A Cytological Diagnosis. *JCDR.* 2017 [cited 2023 Jan 6];ED18&ED20. 10.7860/JCDR/2017/28184.10099 Reference Source 28764182 PMC5535375

[8] Carvalho Godoy FAD Faustino CAC Meneses CS : LOCALIZED PIGMENTED VILLONODULAR SYNOVITIS: CASE REPORT. *Revista Brasileira de Ortopedia (English Edition).* 2011 Jul;46(4):468–471. 10.1016/S2255-4971(15)30264-0 27027040 PMC4799282

[9] Luo D Yu L Yang L : Atypical and bilateral pigmented villonodular synovitis of wrist in an adolescent patient: case report and literature review. PMC7994141 33786156

[10] AlDakhil M AlDakhil A Karami M : Atypical presentation of pigmented villonodular synovitis: A case report. *Saudi J Med Med Sci.* 2019;7(1):44–46. 10.4103/sjmms.sjmms_180_16 30787857 PMC6381854

[11] Carpintero P Serrano J García-Frasquet A : Pigmented villonodular synovitis of the wrist invading bone--a report of 2 cases. *Acta Orthopaedica Scandinavica.* 2000 Jan;71(4):424–426. 10.1080/000164700317393475 11028896

[12] Gupta S Mishra RS : Cytologic Appearance of Pigmented Villonodular Synovitis. *Acta Cytologica.* 2002;46(4):728–730. 10.1159/000326984 12146040

[13] Sitati FC : Pigmented villonodular synovitis of the knee: a case report. 10.1016/j.jus.2011.02.001 PMC3558236 23396820

