# Supplementary material for: Case Report: Cytodiagnosis of pigmented villonodular synovitis involving carpal bones of right wrist
Source: F1000Res. 2023 Nov 21;12:1490. [Version 1] doi: 10.12688/f1000research.141797.1 (PMC11217717; doi:10.12688/f1000research.141797.1)
Supplement: Peer review history

# Reviewer report: Reviewer response for version 1

**DOI:** 10.5256/f1000research.155275.r293430

**Published:** 2024-07-01

**Authors:** Priyambada Kumar

**Affiliations:** 1 Bombay Hospital and Medical Research Centre, Mumbai, Maharashtra, India

Thank you for giving me the opportunity to review this article. It is well written and reflects the rare presentation and diagnostic challenges associated with PVNS.

 I would just like to add the following few comments which may be taken into consideration for correction:

 Comment 1:Diagnostic Tests- The blood tests, X-ray, MRI, FNAC, and biopsy results are comprehensive and well-documented. Ensure the narrative connects these findings logically.

 Comment 2: The discussion provides a good overview of PVNS, including typical presentations and the rarity of this case. It is advisable to compare this case to other rare presentations of PVNS more explicitly. I suggest for inclusion of more references to recent literature citing rare cases.

 Apart from these minor suggestions, I think the article is fit to be indexed in its present format.

Are enough details provided of any physical examination and diagnostic tests, treatment given and outcomes?

Yes

Is the case presented with sufficient detail to be useful for other practitioners?

Yes

Is sufficient discussion included of the importance of the findings and their relevance to future understanding of disease processes, diagnosis or treatment?

Yes

Is the background of the case’s history and progression described in sufficient detail?

Yes

Reviewer Expertise:

orthopaedics, orthopaedic spine surgery

I confirm that I have read this submission and believe that I have an appropriate level of expertise to confirm that it is of an acceptable scientific standard.

# Reviewer report: Reviewer response for version 1

**DOI:** 10.5256/f1000research.155275.r236047

**Published:** 2024-04-19

**Authors:** Neelkamal Kapoor

**Affiliations:** 1 All India Institute of Medical Science, Bhopal, Madhya Pradesh, India

It is a well written case report describing a comparatively rare entity which is found in an unusual location. Also it is unusual to diagnose it with the help of cytology. Pigmented Villonodular Synovitis is sometimes associated with other morbidities of bones and joints and also is prone to recurrence thus this case report is an important addition to the existing literature specially from the patient care perspective. (Reference number 13 is incomplete).

Are enough details provided of any physical examination and diagnostic tests, treatment given and outcomes?

Yes

Is the case presented with sufficient detail to be useful for other practitioners?

Yes

Is sufficient discussion included of the importance of the findings and their relevance to future understanding of disease processes, diagnosis or treatment?

Yes

Is the background of the case’s history and progression described in sufficient detail?

Yes

Reviewer Expertise:

Surgical Pathology.

I confirm that I have read this submission and believe that I have an appropriate level of expertise to confirm that it is of an acceptable scientific standard.
